# Separating signal from noise: the challenge of identifying useful biomarkers in sepsis

**DOI:** 10.1186/cc13770

**Published:** 2014-03-17

**Authors:** Russell J McCulloh, John A Spertus

**Affiliations:** 1 Division of Pediatric Infectious Diseases, Children’s Mercy Hospital, 2401 Gillham Road, Kansas City, MO 64108, USA; 2 University of Missouri-Kansas City School of Medicine, 2411 Holmes Road, Kansas City, MO 64198, USA; 3 Saint Luke’s Mid America Heart Institute, 4401 Wornall Road, Kansas City, MO 64111, USA

## Abstract

Sepsis diagnosis remains based largely on clinical presentation despite significant advances in the understanding of underlying pathophysiology and host-pathogen interactions. The systematic review article by Zonneveld and colleagues in the previous issue of *Critical Care* describes another potential avenue of study for using biomarkers for sepsis diagnosis and prognostication. Soluble leukocyte adhesion molecules and their associated sheddase enzymes vary in detectable levels and activity in patients in relation to immunologic status, age, and systemic inflammation, including in the setting of sepsis. Unfortunately, studies of these molecules as diagnostic or prognostic aids (or both) in sepsis have thus far been disappointing. Zonneveld and colleagues propose two potential avenues to enhance the performance characteristics of soluble adhesion molecules and their sheddases in sepsis diagnosis and prognosis: (a) identifying age-adjusted normal values for soluble leukocyte adhesion molecules and their sheddases and (b) investigating simultaneous measurement of both soluble adhesion molecules and sheddases in integrated sepsis evaluation schema. This commentary discusses the proposed solutions of Zonneveld and colleagues in more detail and outlines additional considerations that should be addressed in order to develop robust and valid diagnostic and prognostic tools for clinicians managing patients with sepsis.

## Introduction

The article by Zonneveld and colleagues [1] in the previous issue of *Critical Care* highlights the ongoing challenge in identifying and treating septic patients. Few human maladies prove more resistant to modern diagnostic and theragnostic techniques than sepsis. Despite research describing much of the underlying pathophysiology, sepsis remains a diagnosis based primarily on clinical presentation [2]. Many attempts at identifying biomarkers that aid in diagnosis or mortality risk estimation have met with limited success [3]. The elusiveness of reliable biomarkers in sepsis stems partly from the byzantine, interdependent immune processes contributing to the sepsis syndrome. Zonneveld and colleagues review the complex interplay of one set of processes. Leukocyte adhesion molecules, and the sheddase enzymes catalyzing the release of their soluble fragments into the bloodstream, participate in the homeostasis of immune responses that direct leukocytes to sites of injury, infection, and/or inflammation. The authors review previous failed attempts to use soluble adhesion molecules and sheddases as diagnostic biomarkers. Undaunted by the litany of failures identified in their review, Zonneveld and colleagues describe two key avenues of inquiry that may reveal the usefulness of soluble leukocyte adhesion molecules and their sheddases in sepsis diagnosis and prognosis.

## Proposed avenues of inquiry

First, the authors posit that understanding the variation in circulating levels of soluble adhesion molecules and sheddases by patient age will help better define normal values in neonatal, pediatric, and adult sepsis. More accurately defining these normal ranges could improve the diagnostic and prognostic accuracy of these molecules in sepsis. To support this assertion, the authors review several studies describing developmental differences in circulating levels of soluble adhesion molecules and sheddase activity between neonates, children, and adults. The authors’ findings are similar to published studies of other age-related differences in aspects of the immune system. For example, CD4^+^ T-cell counts based on patient age (for example, a Z-score) better predict opportunistic infection risk in HIV-positive children than absolute CD4 counts [4,5]. Age-stratified values have also been suggested for evaluating the use of sepsis biomarkers in older patients [6].

Second, Zonneveld and colleagues propose that measuring soluble adhesion markers and their sheddases simultaneously may provide greater diagnostic and prognostic capabilities than measuring either category separately. Their assertion is important because the complex, inter-related nature of the immunologic response to sepsis most likely cannot be distilled to a single component. Prognosis is undoubtedly related to numerous factors, including biomarker-associated measures of sepsis severity, age, comorbidities, disease severity (for example, oxygenation, organism, or etiology of the septic episode), and other factors contributing to mortality. Evaluation strategies thus should seek to integrate the contributions of multiple potentially prognostic elements.

The challenge, therefore, is to develop a research strategy that provides the infrastructure capable of modeling the heterogeneity of patient outcomes. The literature reviewed by Zonneveld and colleagues is woefully inadequate to do this. Few of the studies report the association of biomarker changes to outcome, and all are limited by very small sample sizes. Ideally, researchers need to assemble a large, multi-center cohort that exquisitely characterizes septic patient demographics, medical comorbidities, disease severity, and sepsis-associated biomarkers. The PROGRESS (Promoting Global Research Excellence in Severe Sepsis) registry represents an attempt to create such a cohort but was limited by variation in reporting rates and lack of data integrity assurance measures [7]. Most importantly, the ideal registry needs to accurately document patient outcomes. From such an observational registry, researchers could build multi-variable models of mortality, in which the independent prognostic importance of each variable could be determined, along with any possible interactions. In other disciplines, such as cardiology or clinical trials, such multi-variable models have been invaluable in modeling the heterogeneity of risk and treatment benefits and can be used to guide therapy and improve outcomes [8-12].

Accomplishing this goal will require substantial commitment and collaboration among those interested in building better sepsis prognostic strategies. If, as a simple rule of thumb, 10 mortality events are required for each potential predictor variable and 20% of patients with sepsis die, then for 10 potential predictors (not including interactions) to be identified, a minimum of 500 patients with severe sepsis would need to be enrolled to perform such an analysis, which represents a major effort. This commitment is critical, however, to developing tools and strategies to improve prognostication, incorporate evolving insights into sepsis pathophysiology, and use this information to better tailor therapy and improve outcomes.

## Conclusions

We commend Zonneveld and colleagues for their systematic review of the leukocyte adhesion molecule-sheddase system in sepsis. Their work highlights the needs for more sophisticated methods to define prognosis and for challenging the research community to be sensitive to potential age-based interactions. The job for researchers and policymakers now is to invest in collecting the data from large, multi-site cohorts and to rigorously analyze the information so that better tools for prognostication can be developed. Once available, these tools can become foundational for better informing patients and families about prognosis, improving diagnostic testing, and directing treatments to those who most benefit.

## Competing interests

The authors declare that they have no competing interests.
